# QTL and QTL networks for cold tolerance at the reproductive stage detected using selective introgression in rice

**DOI:** 10.1371/journal.pone.0200846

**Published:** 2018-09-17

**Authors:** Yuntao Liang, Lijun Meng, Xiuyun Lin, Yanru Cui, Yunlong Pang, Jianlong Xu, Zhikang Li

**Affiliations:** 1 Institute of Crop Sciences, Chinese Academy of Agricultural Sciences, Beijing, China; 2 Rice Research Institute, Guangxi Academy of Agricultural Sciences, Nanning, China; 3 Agricultural Genomics Institute at Shenzhen, Chinese Academy of Agricultural Sciences, Shenzhen, China; 4 Rice Research Institute, Jilin Academy of Agricultural Sciences, Jilin, China; New Mexico State University, UNITED STATES

## Abstract

Low temperature stress is one of the major abiotic stresses limiting the productivity of *Geng* (*japonica*) rice grown the temperate regions as well as in tropical high lands worldwide. To develop rice varieties with improved cold tolerance (CT) at the reproductive stage, 84 BC_2_ CT introgression lines (ILs) were developed from five populations through backcross breeding. These CT ILs plus 310 random ILs from the same BC populations were used for dissecting genetic networks underlying CT in rice by detecting QTLs and functional genetic units (FGUs) contributing to CT. Seventeen major QTLs for CT were identified using five selective introgression populations and the method of segregation distortion. Of them, three QTLs were confirmed using the random populations and seven others locate in the regions with previously reported CT QTLs/genes. Using multi-locus probability tests and linkage disequilibrium (LD) analyses, 46 functional genetic units (FGUs) (37 single loci and 9 association groups or AGs) distributed in 37 bins (~20%) across the rice genome for CT were detected. Together, each of the CT loci (bins) was detected in 1.7 populations, including 18 loci detected in two or more populations. Putative genetic networks (multi-locus structures) underlying CT were constructed based on strong non-random associations between or among donor alleles at the unlinked CT loci/FGUs identified in the CT ILs, suggesting the presence of strong epistasis among the detected CT loci. Our results demonstrated the power and usefulness of using selective introgression for simultaneous improvement and genetic dissection of complex traits such as CT in rice.

## Introduction

As the staple food for the half world population, rice (*Oryza sativa* L.) grows worldwide. There are two major subspecies of *O*. *sativa*, *Xian* (*indica*) or *Geng* (*japonica*) [1], the former of which is widely grown in the tropical and subtropical areas, while the latter in the temperate areas of high latitude/altitude of Asia. For *Geng* rice crops grown primarily in high-latitude or high-altitude regions of China, Japan, Korea, and other parts of the world, low temperature, or cold stress, is one of the most common environmental stresses affecting rice growth and development, and thus grain yields. Cold stress includes two major classes, chilling (0–15°C) and cold deep-water irrigation (CDWI) (18–19°C) stress, both of which are major environmental factors limiting rice productivity [2,3]. The former occurs primarily at early stages of rice crops, causing poor crop establishment and delayed development, while the latter causes low fertility and poor grain filling. Thus, developing cold tolerant (CT) rice varieties is the most efficient way to resolve the problem.

CT in rice is a quantitative trait controlled by multiple genes and involving complex biological and genetic mechanisms. To date, QTL mapping using bi-parental or multiple cross populations identified more than 250 QTLs affecting CT at different development stages of rice [3–10]. Mapping QTL for CT at the booting stage is more difficult because of difficulties in phenotyping. Of these, 108 QTLs for CT were detected at the booting stage (S1 Table). Four QTLs, *qCT8*, *qCTB7*, *qCTB3* and *qCT-3-2*, have been fine mapped [6, 11–13], *qCTB10-2* was delimited to a 132.5 kb region containing 17 candidate genes and 4 genes were cold-inducible [14], and only two CT QTL, *Ctb1* and *CTB4a*, have been cloned and functionally characterized [15,16]. *Ctb1* encodes an F-box protein and functions as part of the E3 ubiquitin ligase complex that is associated with increased spikelet fertility (SF) under cold stress [11]. *CTB4a* encodes a conserved leucine-rich repeat receptor-like kinase [16]. Despite the progress in genetic and molecular dissection of CT in rice, few of these mapped or cloned CT QTL have been used in breeding because of the possible epistasis and QTL x environment interactions [17]. In contrast, breeding progress for improving CT in *Geng* rice remains slow by the conventional pedigree method. This is because almost all crosses have been made between different varieties of the same *Geng* subspecies, which is known to have very low genetic diversity [18]. Breeders have also been reluctant to use *Xian* varieties as donors because of the genetic barriers such as hybrid sterility and hybrid breakdown commonly seen in *Xian*-*Geng* crosses.

To overcome the problem of breeding and genetic research being separate activities in the past study, we have been practicing a strategy of simultaneous improvement and genetic dissection of complex traits by selective introgression, which have been demonstrated to be powerful in several successful applications [19–27]. This strategy includes two parts: (1) development of large numbers of trait-specific introgression lines (ILs) by backcross (BC) breeding; (2) genetic and molecular dissection of target traits using the ILs and marker-based tracking. In this study, we report the application of selective introgression in characterizing the genetic networks underlying CT at the reproductive stage in rice, demonstrating clear advantages of selective introgression over the classical QTL mapping method for simultaneous improvement and genetic dissection of complex traits.

## Materials and methods

### Plant materials

The materials used in this study included two sets of introgression lines (ILs) from five BC_2_F_4_ populations derived from crosses between Chaoyou1 (CY1, the recipient), a high yield *Geng* variety, and 5 donors (3 *Xians* and 2 *Gengs* from China, Vietnam, Nepal and India) (Table 1). The first set of ILs consisted of 84 BC_2_F_4_ ILs developed by two rounds of selection for cold tolerance (CT) at the reproductive stage under cold water, as described previously [22]. The second set of ILs included 310 random ILs developed by single-seed decent from the same five BC_2_ populations without any selection.

**Table 1:** Spikelet fertility (SF) of 84 introgression lines (ILs) under cold water irrigation selected from 5 Chaoyou 1 (CY1, the recipient) BC_2_ populations in 2008, 2009 and 2010.

				Spikelet fertility (%) under cold water ^1^		
Donor (code)	Subspecies	Origin	No. of	2008	2009	2010
			genotypes	Mean ± SD	Mean ± SD	Mean ± SD
X22 (A)	*Xian (I)*	Vietnam	15	64.0±10.8	62.1±9.8	43.8± 19.2
Yuanjing7 (B)	*Geng (J)*	China	15	73.2±10.3	76.4±4.4	34.2±5.1
Fengaizhan (C)	*Xian (I)*	China	22	77.8±11.4	78.9±4.7	59.6±8.5
Chhomrong (D)	*Geng (J)*	Nepal	15	77.7±8.1	69.2±10.6	48.0±16.2
Doddi (E)	*Xian (I)*	India	17	74.4±8.8	67.4±8.8	37.6±10.6
Mean				73.8	71.4	46.0
CY1 (CK)	*Geng (J)*	China		24.8	35.1	10.5

^1^ 2008SF: preliminary single plant screening (spikelet fertility> 50%) of the BC_2_F_4_ bulk populations; 2009SF: progeny testing for spikelet fertility of the selected BC_2_F_5_ lines; 2010SF: second round progeny testing of BC_2_F_6_ lines for spikelet fertility.

**Fig 1:**
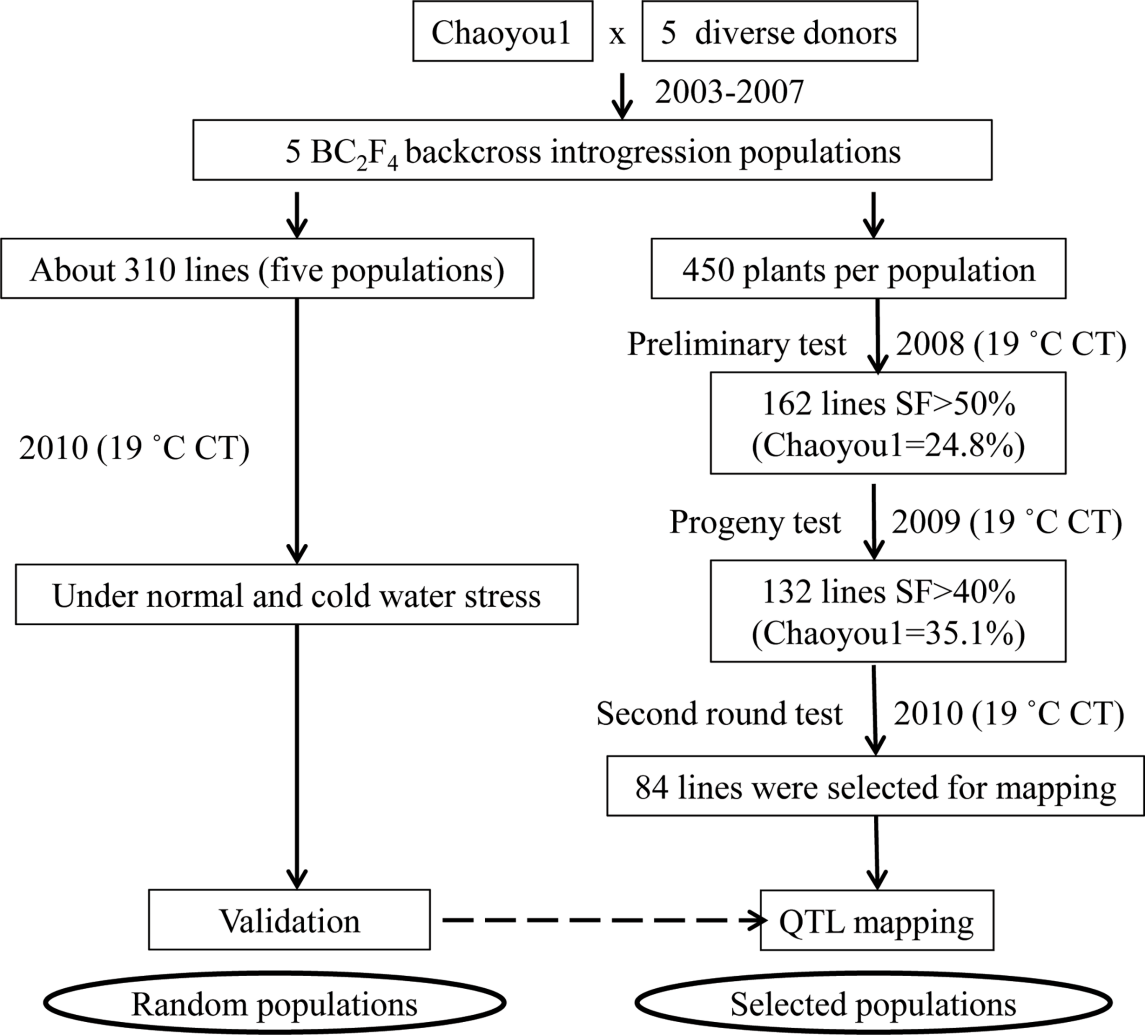
The population development for identification and validation of QTL for cold tolerance in rice.

Fig 1 shows the procedure for developing the two sets of ILs. Briefly, seeds of the five BC_2_F_4_ bulk populations were sown in the seedling nursery on April 15, 2008, and 450 40-day seedlings of each BC_2_F_4_ bulk population were transplanted into a 45-row plot at the Rice Research Institute of Jilin Academy of Agricultural Sciences (JAAS). When the RP (CY1) entered the panicle initiation stage, the cold-water treatment was initiated by constant irrigation of cold water (19± 0.5°C). The cold-water depth in the pond was 20 cm and the treatment was maintained for ~30 days until panicles of almost all plants exerted completely. Then, irrigation with normal temperature water was resumed until the maturity. Under this cold-water treatment, CY1 had spikelet fertility (SF) of 24.8±4.3%, then, 162 BC_2_F_4_ plants with SF>50% were selected. The selected 162 BC_2_F_5_ introgression lines (ILs) from the five populations were progeny tested under the same condition in 2009. Under this cold-water treatment, CY1 had a mean SF of 35.1±3.5%, then, 132 lines with SF>48% were selected. Further progeny testing of the 132 CT ILs under the cold water treatment in 2010 resulted in final selection of 84 CT ILs (SF>40%) from the five populations were used for genotyping and genetic dissection of CT. Performance of ILs and recipient parent (Chaoyou1, CY1) for SF during the first two rounds of selection under cold water stress were shown in S2 Table.

### Phenotyping of the random populations for SF and yield related traits under cold water stress and non-stress conditions

In the summer of 2010, the phenotyping experiments of the two sets of ILs were conducted in two locations, Beijing and Jilin. On 15 April 2010, seeds of the 412 ILs (including 132 CT ILs and 310 random ILs) were sown in the seedling nursery and 15 40-day-old seedlings of each IL were transplanted into a single-row plot the fields in the sheltered pond of JAAS. The field experiment was randomized with two replications and CY1 was inserted in every 50 lines as the check. The LT treatment using cold water was the same as in 2008 and 2009. At maturity, eight representative plants in the middle of each plot were sampled and then eight main panicles of each sampled plant were measured for spikelet number per plant, filled grain number per plant and SF. Under this cold water treatment, the CY1 had a mean SF of 10.5%. In Beijing, seeds of all ILs and CY1 were sown on May 5, and 15 30-day old seedlings of each IL were planted into a one-row plot with a spacing of 25 × 15 cm. The field arrangement was the same as in the LT treatment. Heading date (HD, in days) was visually recorded for all plots when ≥50% of the plants in a plot had headed. At the maturity, five plants of each plot under the cold-water and normal conditions were evaluated for plant height (in cm), panicle length (in cm), spikelet number per panicle (SN), filled grain number per panicle (GN), the spikelet fertility (SF = 100 × GN/SN, in %), panicle number per plant (PN), 1000-grain weight (GW, in g), biomass (BM, in g) per plant, grain yield per plant (GY, in g) and harvest index (HI) in the same way as described previously [22].

### Genotyping of the selected and random ILs

In the 2010 cold treatment experiment when CY1 had SF of 10.5%, 84 BC_2_F_6_ ILs with SF>30% and the 310 random BC_2_F_4_ ILs (ranging from 59 to 65 per population) were used for genotyping (Table 1). More than 600 anchor SSR markers covering the whole rice genome from the Grammene database (Grammene, http://www.gramene.org) were used to survey the polymorphism between CY1 and the donors, from which an average of 113 well-distributed polymorphic SSR markers were used to genotype the selected and random ILs. Marker positions and genetic distances between linked markers were based on the Cornell SSR maps (Grammene, http://www.gramene.org), which covered all 12 rice chromosomes with a total genome size range from 1,125.0 cM in population CY1/Yuanjing7 to 1649.8 cM in population CY1/Chhomrong, with average genetic distances between adjacent markers ranging from 15.2 to 23.7 cM.

### Data analyses

Three different approaches were used to detect QTL and genetic networks underlying CT that were segregating in the five populations. First, we used the segregation distortion method to detect main-effect QTLs in the selected IL populations. This method takes advantage of several populations together and performs a joint analysis to detect QTL that are respond to directional selection in multiple populations based on a consensus map [28]. We also used the traditional method to verify CT QTL by detecting marker-SF associations in the random ILs based on *t*-tests. In this method, we used the minimum threshold of P<0.05 because of the small population sizes of the random populations. The third methodology was more comprehensive and was used to detect QTL and QTL networks in three steps based on the molecular quantitative genetics theory [3, 29]. In this theory, two concepts, functional genetic units (FGUs) and the principle of hierarchy, are defined here based on two commonest types of functional relationships between genes acting in the same signaling pathways affecting complex traits. In the selection experiment, alleles at segregating regulatory loci affecting the target trait (CT) were expected to respond more strongly than their regulated downstream ones, and show greater shifts in their introgression frequencies (IF) of the functional genotypes (FG) in response to selection. Here, FG was defined as one of the two homozygotes and heterozygote that contain the functional allele at involved loci. Based on the concepts, a FGU could be either single loci showing significant excess introgression or an association group (AG) of *r* (*r*≥2) unlinked but perfectly associated loci of equal introgression in CT ILs selected from each BC population. Two types of statistical tests were performed to detect FGUs associated with ST. First, *X*^*2*^ tests were performed to detect whether the allelic and genotypic frequencies at individual loci across the genome in ST ILs from each BC population deviated significantly from the random unselected populations. Second, a multi-locus probability test, *P*_(*AG*)_ = (*p*_*i*_)^*rm*^ • (1−*p*_*i*_)^*r*(*n*−*m*)^, was used to detect individual AGs for CT, where *p*_*i*_ is the frequency of the donor introgression in the random ILs from each BC population, *n* is the number of the selected ILs, *m* is the number of ILs that have co-introgression of the donor alleles, and (*n*−*m*) is the number of ILs having no introgression at the *r* unlinked loci in the AG. Here (*P*_*i*_)^*m*^ is the probability of *m* ILs having co-introgression of the donor alleles and (1-*P*_*i*_)^*n*-*m*^ is the probability of (*n*-*m*) ILs having no introgression at *r* unlinked loci. The threshold to claim a significant case was *P* ≤ 0.005 for individual cases. For any detected AG of *r* (*r* ≥ 2) perfectly associated loci, there will be *r·*(*r–*1)/2 significant pairwise associations between the *r* loci, which were also confirmed by the linkage disequilibrium (LD) analyses [30]. Because each of the BC populations were related to other populations by sharing the same recipient, we also listed putative ST loci detected with the sub-thresholds of 0.05 < P < 0.005 if they were detected with the selected threshold in one or more related populations.

To reveal the multi-locus structure of the detected FGUs–the putative genetic network underlying ST in the ILs from each BC population, pairwise gametic LD analyses were performed to characterize the relationships between alleles at all ST FGUs detected in ILs from each BC population [3, 29]. The LD statistics [26], ${\hat{D}}_{AB}={\tilde{p}}_{AB}-{\tilde{p}}_{A}{\tilde{p}}_{B}$, were calculated using the genotypic data of ILs from each BC population, where ${\tilde{p}}_{AB}$, ${\tilde{p}}_{A}$ and ${\tilde{p}}_{B}$ were the frequencies of co-introgressed functional genotype AB, and functional genotypes at FGUs A and B, respectively. Then, a multi-locus genetic network containing all FGUs detected in the ILs selected from each BC population was constructed in the following 2 steps based on the principle of hierarchy: 1) all FGUs detected in the ILs from a single population were divided based on the LD results into major groups such that individual FGUs of different introgression frequencies within each group were all significantly and positively associated with $\hat{D}s_{AB}{}_{}^{'}$ = 1.0 (*p* ≤ 0.05), and FGUs in different groups were either independent, or negatively associated; and 2) all associated FGUs within each group were connected, forming multiple layers, according to their progressively reduced FG frequencies and inclusive relationships [3].

## Results

### Performances of selected and random ILs under cold water and normal conditions

When compared with the normal conditions, the cold-water stress of 2010 caused delayed heading by 14 days and greatly reduced trait values of CY1 for all measured traits (Table 2). This trait reduction was more pronounced for SF (by 71.9%), GNP (90.8%) and HI (58.4%) than for PN (38.6%), PH (7.9%), SN (27.9%) and GW (34.1%). In contrast, the selected ILs from each of the five populations had significantly improved CT by average 23.7% increased SF for ILs from CY1/Yunjing7 (the most closely related *Geng* donor to CY1) up to 49.1% increased SF for ILs from CY1/Fengaizhan (*Xian*). The average yield advantage over CY1 ranged from 200% for ILs from populations CY1/Doddi and CY1/X22 to 400% for those from population CY1/Fengaizhan. This improved CT was also associated with an average 36.7% increased biomass, 287% increased number of filled gains/panicle, 35.9% increased harvest index, 11.8% increased height, 9.1% increased grain weight, and 4% delayed heading under the cold-water stress. Under the non-stress conditions, the selected ILs showed same trait values as CY1 except for significantly increased height and reduced HI by 12.7cm and 3.4%, as well as slightly increased SN and delayed heading by 11.1 spikelets/panicle and 3 days. In particular, those selected ILs from population CY1/Fanaizhan had apparently better CT than those from other populations, while ILs from Yunjing7 showed the lowest level of CT.

**Table 2:** Summary statistics of five random backcross populations (BC_2_F_6_) for spikelet fertility (in %) evaluated under the cold water stress and normal control conditions in 2010.

Donor parent	No. of lines	Control		Cold water		Difference	
		Mean ± SD	Range	Mean ± SD	Range	Mean ± SD	Range
X22	63	70.7±10.6	33.0–87.6	3.5±4.1	0–16.9	67.4±11.2	25.7–86.8
Yuanjing7	65	72.2±7.0	45.4–82.5	15.6±10.1	2.9–46.7	56.6±11.1	26.5–75.7
Fengaizhan	59	65.3±12.7	33.1–93.7	2.8±3.9	0–13.9	62.5±13.2	20.3–93.7
Chhomrong	64	76.3±8.7	46.8–93.0	7.9±7.7	0–28.6	68.5±10.4	44.0–93.0
Doddi	59	66.3±13.6	28.9–85.3	8.7±9.5	0–38.4	57.6±15.9	19.3–84.1
Chaoyou1		82.4		10.5		71.9	

However, ILs from each of the random populations showed significantly lower SF than CY1 under both the normal and cold-water conditions, except for ILs from populations CY1/Yunjing7 and CY1/Chhomrong derived from parents of the same *Geng* subspecies (Table 2). Thus, the reduced fertility in the random progenies from the three inter-subspecific crosses was commonly seen in most breeding populations of this type [31]. Also, ILs from each population showed considerable variation in SF under both the normal and cold water conditions. The CT selected ILs had an average 9.3% of the donor genomes, significantly less than the expected 12.5% for BC_2_ populations, and ranged from 4.7% in those of population CY1/Yunjing7 to 13.1% in the ILs of population CY1/Chhomrong. The average heterozygosity of the selected ILs was 1.9%, ranging from 1.0% in those of population CY1/Yunjing7 to 4.4% in the ILs of population CY1/X22. In contrast, the random ILs showed slightly less donor introgression (8%) but higher heterozygosity at 2.9%, when compared to the selected ILs.

### Detection of CT QTL in selected ILs

**Fig 2:**
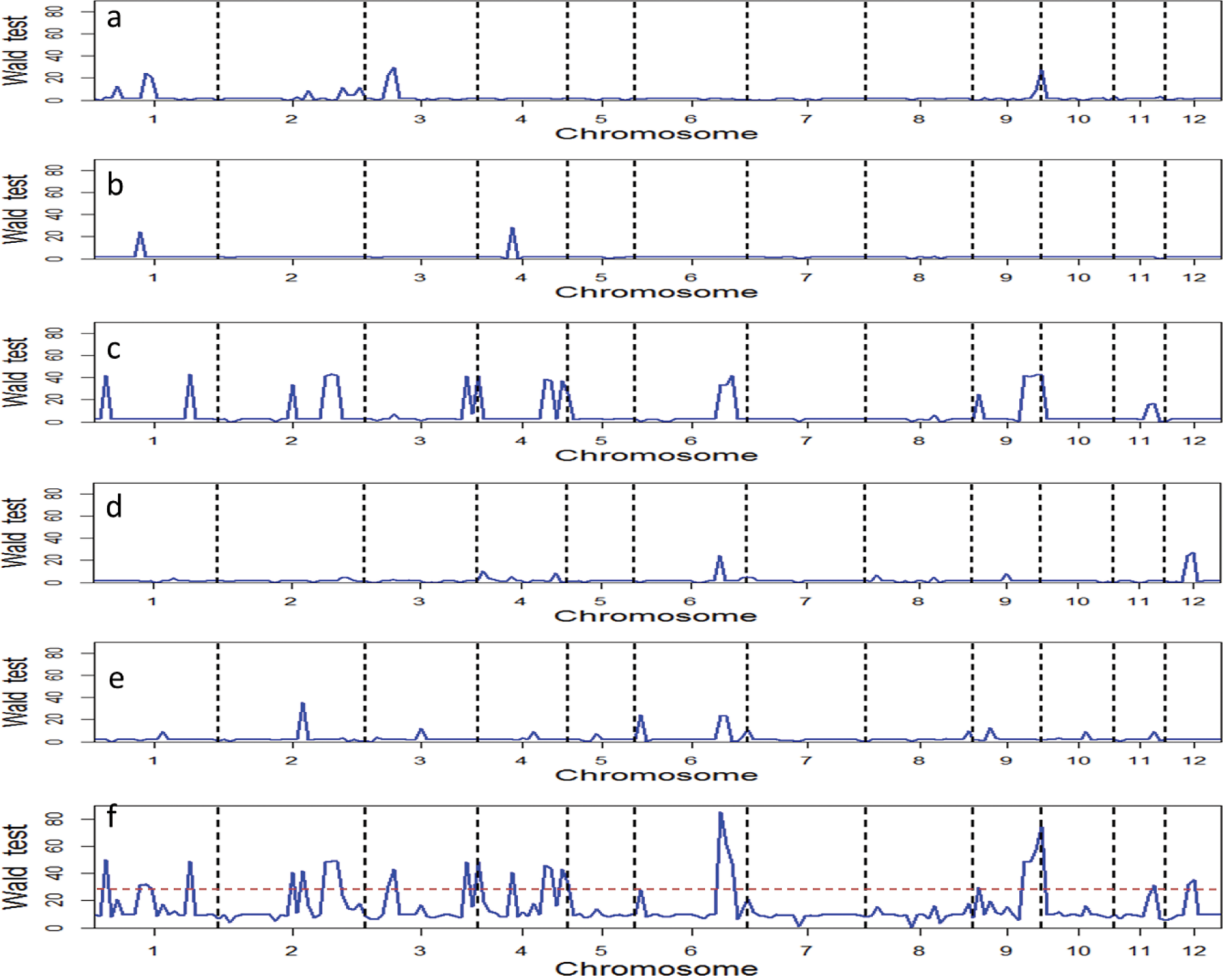
Wald tests of single and combined five populations selected for spikelet fertility under cold water stress: a): population CY1/X22 (A); b): population CY1/Yuanjing7 (B); c): population CY1/ Fengaizhan (C); d): population CY1/ Chhomrong (D); e): population CY1/ Doddi (E); f): population combined five populations. The horizontal broken line indicated that Wald value is 28.0 threshold at P value of 0.01.

Based on the segregation distortion in single populations, we detected 17 QTLs for CT (SF under cold-water) on rice chromosomes 1, 2, 3, 4, 6, 9, 11 and 12 when five populations were combined using a consensus linkage map (Fig 2, Table 3). These included 3 QTLs in population CY1/X22(A), 2 QTLs in population CY1/Yuanjing7 (B), 11 QTLs in population CY1/Fengaizhan (C), 2 QTLs in population CY1/Chhomrong (D) and 4 QTLs in population CY1/Doddi (E), respectively (Fig 2). Of the 17 QTLs, *qCT1*.*3*, *qCT6*.*7* and *qCT9*.*6* were detected in two of the populations, while *qCT6*.*5* was detected in three populations (Table 3). Three of these CT QTLs (*qCT3*.*12*, *qCT6*.*7* and *qCT9*.*6*) were validated in one or two of the random populations (Table 4).

**Table 3:** Quantitative trait loci (QTL) for rice cold tolerance (CT) detected in five selected introgression populations using single and joint segregation distortion analyses.

QTL	Marker	Wald statistics ^1^						Reference
		Total	A	B	C	D	E	
*qCT1*.*2*	RM522	50.3	2.6	1.8	**41.9**	1.8	2.1	*CRI* [7]
*qCT1*.*3*	RM312	31.8	**24.3**	**24.3**	2.7	1.1	2.2	*qSLT1-1* [8]
*qCT1*.*10*	RM200	49	0.4	1.8	**42.9**	1.6	2.2	
*qCT2*.*4*	RM29	40.5	1.1	1.9	**33.5**	1.9	2.2	*qCTB2a* [9]
*qCT2*.*5*	RM341	41.9	0.5	1.8	2.6	1.8	**35.2**	
*qCT2*.*8*	RM240	48.7	0	1.8	**42.9**	1.8	2.2	*QTL2*.*1* [10]
*qCT3*.*4*	RM7	43.1	**29.8**	1.9	6.7	2.5	2.2	
*qCT3*.*12*	RM227	48.8	1.7	1.7	**41.9**	1.7	2	
*qCT4*.*4*	RM185	40.5	1.8	**28.6**	2.6	5.3	2.2	
*qCT4*.*6*	RM317	45.8	1.8	1.8	**38.5**	1.4	2.2	*Ctb-1* [15]
*qCT6*.*1*	RM508	28.1	0.9	1.7	0.2	0.6	**24.5**	
*qCT6*.*5*	RM162	85.4	1.8	1.8	**33.6**	**24.4**	**23.7**	*qLTSSvR6-2* [4]
*qCT6*.*7*	RM400	61.2	0.7	1.9	**33.4**	1.8	**23.4**	
*qCT9*.*1*	RM41	29.9	0	1.8	**24.9**	1.8	1.4	
*qCT9*.*6*	RM160	74.9	**27.5**	1.8	**42.4**	1.8	1.4	
*qCT11*.*5*	RM457	31.3	1.6	1.7	**16.9**	1.8	9.3	*CRI* [7]
*qCT12*.*4*	RM519	35.6	1.8	1.8	2.5	**27.4**	2.1	

^1^ The Wald statistics values of P ≤ 0.05 and 0.01 = 22.2 and 28.0, respectively.

**Table 4:** Detection of QTL, via *t*-tests, affecting cold tolerance associated with spikelet fertility (%) under cold water in five random BC_2_F_4_ populations.

			Genotype			
QTL	Population	marker	AA	BB	Additive effect	*P*-value
*qCT3*.*12*	C	RM148	1.2±2.5	4.1±4.8	1.5	0.01
*qCT3*.*12*	D	RM227	7.7±7.2	14.2±8.2	3.3	0.03
*qCT6*.*7*	E	RM400	7.4±7.2	13.5±13.6	3.1	0.03
*qCT9*.*6*	A	RM160	3.2±3.8	11.2±5.2	4.0	0.00
*qCT9*.*6*	E	RM160	7.9±8.3	18.1±14.7	5.1	0.01

### Genetic networks (multi-locus structures) underlying CT

**Fig 3:**
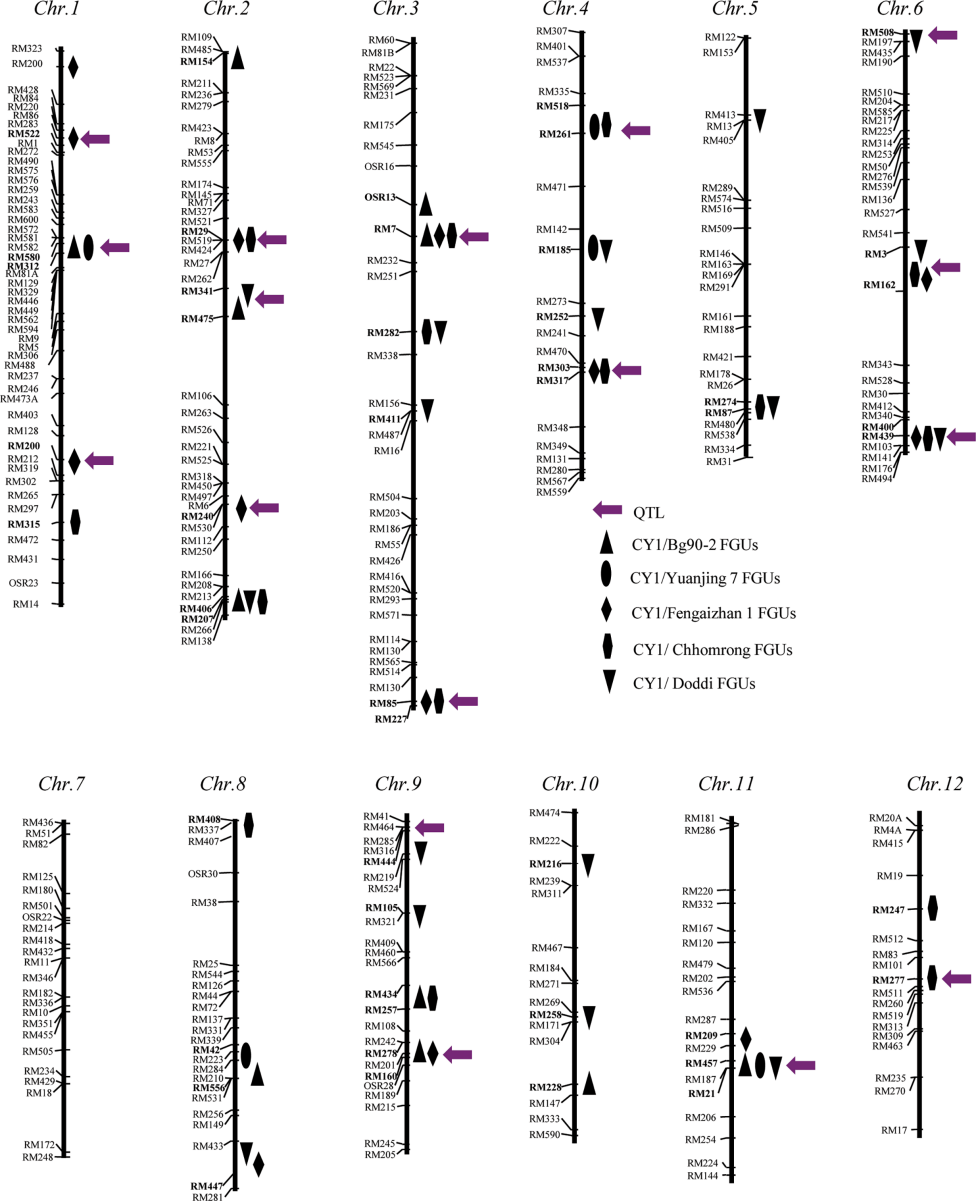
Genomic regions harboring FGUs and QTLs underlying rice cold tolerance at the reproductive stage detected in 84 introgression lines (ILs) from 5 BC_2_F_4_ populations (Tables 2 and 4), in which regions pointed by purple arrows are QTLs detected in the random populations.

Total of 46 functional genetic units (FGUs) (37 single loci and 9 association groups or AGs) for CT were detected by χ^2^ tests (single loci) and multi-locus linkage disequilibrium analyses in 84 cold-tolerant introgression lines (ILs) selected from five populations (Fig 1) and distributed on all 12 chromosomes except chromosome 7 (Fig 3). The number of detected CT loci ranged from 6 loci in 4 FGUs in the CY1/Yuanjing7 (B) population to 16 loci in 13 FGUs in the CY1/Doddi (E) population. The average IF of the donor alleles at the 58 CT loci was 0.46, or 5.75 times as much as the average introgression in the random populations (Table 5). These FGUs were distributed in 39 bins (~20%) across the rice genome.

**Table 5:** Genomic information for 46 functional genetic units (FGUs) (37 single loci and 9 association groups or AGs) for cold tolerance (CT) detected by χ2 tests (single loci) and multi-locus linkage disequilibrium analyses in 84 cold-tolerant introgression lines (ILs) selected from five populations.

Donor	Code	AG^1^	Branch	Marker	Bin^2^	B^3^	H	IF	P-value
X22	A	*agCT*_*A1*_	*A-1*	RM556	8.4	4	0	0.267	9.3E-15
X22	A	*agCT*_*A1*_	*A-1*	RM257	9.5	4	0	0.267	9.3E-15
X22	A	*agCT*_*A1*_	*A-1*	RM228	10.6	4	0	0.267	9.3E-15
X22	A		*A-2*	RM7	**3.5**	12	0	0.800	1.5E-29
X22	A		*A-2*	RM475	**2.5**	5	0	0.333	1.3E-04
X22	A			RM312	**1.3**	10	0	0.667	4.7E-20
X22	A			RM154	2.1	4	0	0.267	6.1E-03
X22	A			RM207	2.9	5	0	0.333	1.3E-04
X22	A			OSR13	3.4	10	0	0.667	4.7E-20
X22	A			RM160	**9.6**	11	0	0.733	1.4E-24
X22	A			RM21	**11.5**	4	2	0.400	5.8E-05
Yuanjing7	B	*agCT*_*B2*_	*B-2*	RM21	**11.5**	4	0	0.267	5.3E-11
Yuanjing7	B	*agCT*_*B2*_	*B-2*	RM261	4.2	4	0	0.267	5.3E-11
Yuanjing7	B	*agCT*_*B1*_	*B-1*	RM580	**1.3**	10	3	0.767	4.5E-37
Yuanjing7	B	*agCT*_*B1*_	*B-1*	RM518	4.2	13	0	0.867	3.8E-50
Yuanjing7	B			RM42	8.4	4	0	0.267	2.6E-04
Fengaizhan	C	*agCT*_*C1*_	*C-1*	RM240	**2.8**	18	0	0.818	7.3E-42
Fengaizhan	C	*agCT*_*C1*_	*C-1*	RM278	**9.6**	18	0	0.818	7.3E-42
Fengaizhan	C		*C-1*	RM200	**1.10**	19	0	0.864	5.0E-62
Fengaizhan	C		*C-1*	RM522	**1.2**	18	2	0.864	2.1E-57
Fengaizhan	C		*C-1*	RM148	**3.12**	19	0	0.864	5.0E-62
Fengaizhan	C		*C-1*	RM303	**4.6**	16	0	0.727	4.0E-43
Fengaizhan	C		*C-1*	RM162	**6.5**	14	0	0.636	1.9E-32
Fengaizhan	C		*C-1*	RM447	8.6	10	4	0.545	1.1E-23
Fengaizhan	C		*C-1*	RM209	**11.5**	8	3	0.432	7.2E-14
Fengaizhan	C		*C-2*	RM29	**2.4**	14	3	0.705	5.6E-37
Fengaizhan	C		*C-2*	RM7	**3.5**	7	0	0.318	2.7E-07
Chhomrong	D	*agCT*_*D1*_	*D-4*	RM7	**3.5**	4	0	0.267	1.1E-09
Chhomrong	D	*agCT*_*D1*_	*D-4*	RM282	3.6	4	0	0.267	1.1E-09
Chhomrong	D		*D-2*	RM439	**6.7**	5	0	0.333	5.6E-04
Chhomrong	D	*agCT*_*D2*_	*D-2*	RM315	1.11	3	0	0.200	7.3E-15
Chhomrong	D	*agCT*_*D2*_	*D-2*	RM227	**3.12**	3	0	0.200	7.3E-15
Chhomrong	D	*agCT*_*D2*_	*D-2*	RM87	5.8	3	0	0.200	7.3E-15
Chhomrong	D	*agCT*_*D2*_	*D-2*	RM247	12.2	3	0	0.200	7.3E-15
Chhomrong	D	*agCT*_*D3*_	*D-3*	RM207	2.9	6	0	0.400	1.8E-13
Chhomrong	D	*agCT*_*D3*_	*D-3*	RM434	9.5	6	0	0.400	1.8E-13
Chhomrong	D		*D-1*	RM3	**6.5**	10	0	0.667	2.9E-19
Chhomrong	D		*D-1*	RM317	**4.6**	6	0	0.400	5.3E-06
Chhomrong	D		*D-1*	RM408	8.1	4	1	0.300	3.8E-03
Chhomrong	D		*D-1*	RM518	4.2	5	0	0.333	5.6E-04
Chhomrong	D			RM85	**3.12**	6	1	0.433	3.3E-07
Chhomrong	D			RM277	**12.4**	10	0	0.667	2.9E-19
Doddi	E	*agCT*_*E1*_	*E-1*	RM411	3.7	7	0	0.412	9.7E-17
Doddi	E	*agCT*_*E1*_	*E-1*	RM141	**6.7**	7	0	0.412	9.7E-17
Doddi	E		*E-2*	RM433	8.6	7	0	0.412	1.0E-08
Doddi	E	*agCT*_*E2*_	*E-2*	RM282	3.6	5	0	0.294	1.6E-12
Doddi	E	*agCT*_*E2*_	*E-2*	RM216	10.2	5	0	0.294	1.6E-12
Doddi	E	*agCT*_*E3*_	*E-1*	RM406	2.9	5	0	0.294	1.6E-12
Doddi	E	*agCT*_*E3*_	*E-1*	RM405	5.2	5	0	0.294	1.6E-12
Doddi	E		*E-3*	RM341	**2.5**	15	0	0.833	2.1E-44
Doddi	E		*E-3*	RM185	**4.4**	4	0	0.235	9.5E-03
Doddi	E		*E-3*	RM274	5.8	10	0	0.588	9.6E-19
Doddi	E			RM252	4.5	6	0	0.353	2.8E-06
Doddi	E			RM162	**6.5**	10	1	0.618	3.4E-19
Doddi	E			RM444	9.3	7	0	0.412	1.0E-08
Doddi	E			RM105	9.4	5	0	0.294	2.7E-04
Doddi	E			RM258	10.5	6	0	0.353	2.8E-06
Doddi	E			RM21	**11.5**	7	0	0.412	1.0E-08

^1^ AGs are defined as a group unlinked but perfectly associated loci of equal introgression in the selected CT ILs from each BC population, detected by multi-locus probability tests. P-value is the probabilities for the null hypothesis that the genotypic frequencies fit the Mendelian segregation based on single locus *X*^*2*^ tests.

^2^ Bold ones were CT QTLs detected by the segregation distortion approach in Table 2.

^3^ B, H and IF are the frequencies of the donor homozygote, heterozygote and donor introgression in the selected CT ILs.

**Fig 4:**
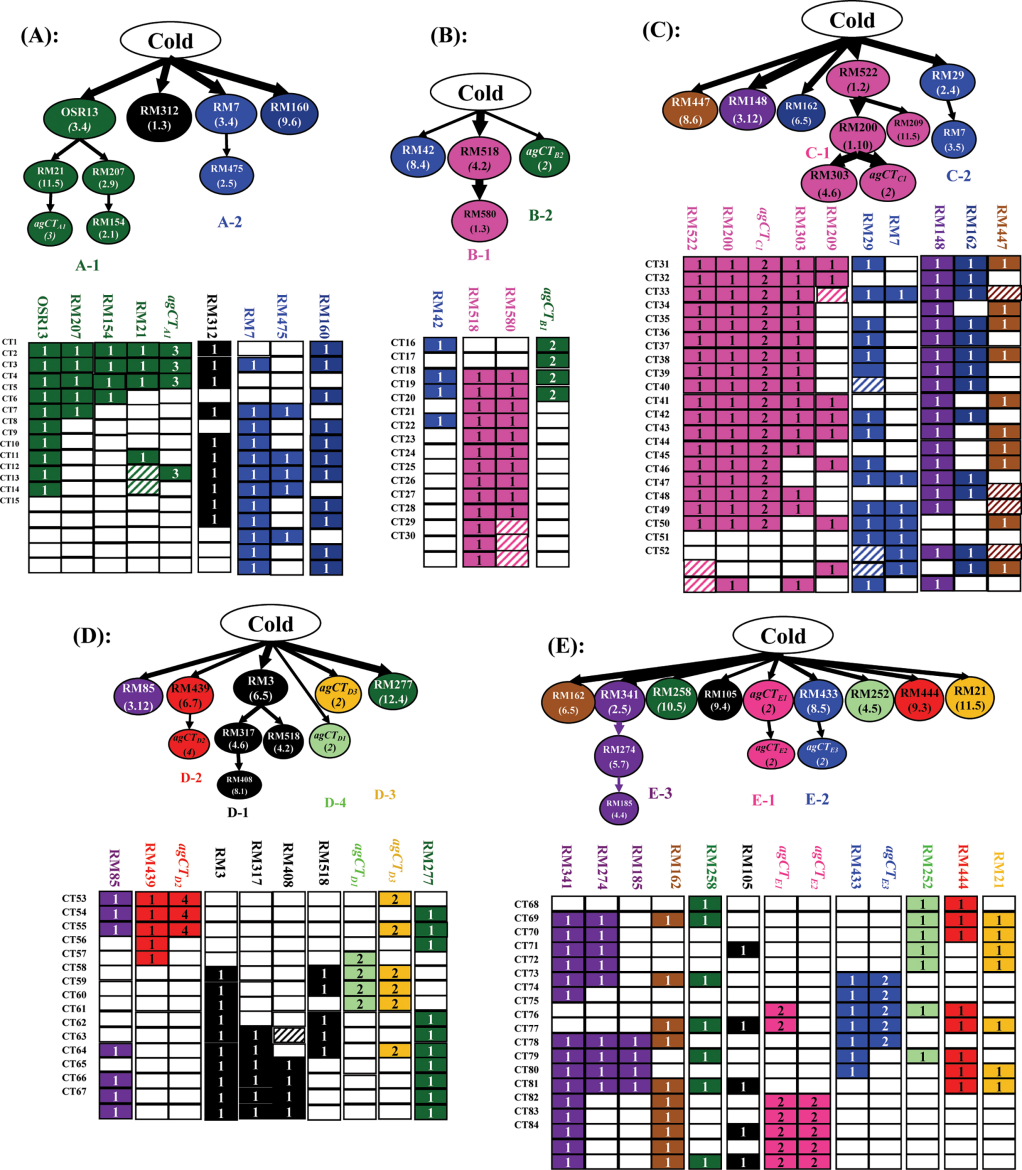
Putative genetic networks (multi-locus structures) underlying cold tolerance (CT) of rice detected in HT backcross introgression lines (BILs) from five (A, B, C, D, E and F) populations. In the corresponding graphical genotypes of each network, the unfilled, fully colored, and patched cells represent the recipient homozygote, donor homozygote, and heterozygote genotypes. The numbers in the cells of each FGU are the number of loci included in the FGUs. The loci (markers) included in each of the detected association groups (AGs) are shown in Table S1. Solid arrow lines connected two FGUs in each branch of a network represent putative functional relationships with those of high introgression as putative regulators in the upstream and those of low introgression in the downstream, and the thickness of an arrow line was proportional to the introgression frequency of the downstream FGU in Table 5.

Fig 4A–4E shows the five putative genetic networks or multi-locus structures each containing all CT FGUs identified in the selected CT ILs from each BC population and their corresponding graphical genotypes at the detected CT FGUs. Fig 4A is the network containing all 9 FGUs (11 loci) detected in the 15 CT CY1/X22 (A) ILs, which has two highly associated groups of FGUs plus 2 independent loci (RM312 in bin1.3 and RM160 in bin9.7). Branch A-1 consisted of two sub-branches with OSR13 (bin 3.4) of high introgression (IF = 0.667) placed in the upstream of the network as the putative regulator. Sub-branch 1 had RM21 (bin11.5) in the upstream and *agCT*_*A1*_ consisting of three unlinked but perfectly associated loci at RM556 (bin8.4), RM257 (bin9.6) and RM228 (bin10.6) in the downstream. Sub-branch 2 had RM207 (bin2.9) in the upstream and RM154 (bin2.1) in the upstream. Fig 4B shows the putative genetic network containing three FGUs (2 AGs and 1 locus) detected in the 15 ILs of the CY1/Yuanjing7 (B) population. Branch B-1 consisted of two unlinked but perfectly associated loci, RM518 (bin4.2) and RM580 (bin1.3). This branch was more important as 13 of the 15 CT ILs had the donor alleles of the loci. Branch B-2 was an AG comprising two unlinked but perfectly associated loci, RM21 (bin11.5) and RM 261 (bin4.3) of relatively low introgression (IF = 0.267). Fig 4C presented the putative genetic network comprising ten FGUs (1 AG and 9 loci) with two major branches plus three independent loci detected in the 22 ILs from population CY1/Fengaizhan. Branch C-1 was the most important one consisting of six unlinked but highly associated FGUs (1 AG and 4 loci) of high introgression. RM522 (bin1.2) was placed at the top of the network as the putative regulator because 20 of the 22 CT ILs had donor alleles at this locus, with the remaining 4 FGUs (RM200 in bin1.10, *agCT*_*C1*_ (bins 2.8 and 9.7), RM303 (bin4.6) and RM209 (bin11.5) in the downstream. The fourth network (Fig 4D) consisted of 11 FGUs (3 AGs and 8 loci) with 4 branches plus two independent loci of high introgression identified in the 15 ST CY1/Chhomrong (D) ILs. The two important independent loci at RM85 (bin3.12) and RM277 (bin12.4) each had an IF of 0.667. Branch D-1 consisted of four unlinked but highly associated loci with RM3 in bin6.5 of high introgression (IF = 0.667) on the top as the putative regulator and three downstream loci of lower introgression at RM317 (bin4.6), RM408 (bin8.1) and RM518 (bin4.2) in the downstream. Branch D-2 consisted of two highly associated FGUs, with RM439 (bin6.7) in the upstream and *agCT*_*D2*_ in the downstream. *agCT*_*D2*_ comprises four unlinked but perfectly associated loci near RM315 (bin1.11), RM227 (bin3.12), RM87 (bin5.8) and RM247 (bin12.2) detected with a P value of 7.3^−15^. Branch D-3 was *agCT*_*D3*_ consisting of two unlinked but perfectly associated loci near RM207 (bin2.9) and RM434 (bin9.5) detected with a P value of 1.8^−13^. Branch D-4 was *agCT*_*D1*_ consisting of two unlinked but perfectly associated loci near RM7 (bin3.5) and RM282 (bin3.6). The fifth network (Fig 4E) consisted of 13 FGUs (3 AGs and 10 loci) in three branches identified in the 17 ST CY1/Doddi (E) ILs. Branch E1 consisted of highly associated FGUs with *agCT*_*E1*_ in the upstream and *agCT*_*E3*_ in the downstream. *agCT*_*E1*_ contained two unlinked but perfectly associated loci near RM411 in bin3.8 and RM141 in bin6.7 detected with a P value of 9.7^−17^, while *agCT*_*E3*_ contained two unlinked but perfectly associated loci near RM406 in bin2.9 and RM405 in bin5.2 detected with a P value of 1.6^−12^. Branch E-2 contained two FGUs with RM433 in bin in the upstream and *agCT*_*E2*_ in the downstream. *agCT*_*E2*_ consisted of two perfectly associated loci near RM282 (bin3.6) and RM216 (bin10.2). Branch E-3 contained 3 unlinked but highly associated loci with RM341 (bin2.5) in the upstream, and RM274 (bin5.8) and RM185 (bin4.4) in the downstream (Table 5, Fig 4E).

Taking above results together, we found the following nine CT QTL were detected in multiple populations and had high introgression (large effect) in the upstream of genetic networks, suggesting these regions may contain putative regulatory genes for CT. These included *qCT1*.*2* (RM532), *qCT2*.*4* (between RM29 and RM341), *qCT3*.*5* (between OSR13 and RM7), *qCT3*.*12* (RM85), *qCT4*.*2* (RM518), *qCT4*.*6* (between RM303 and RM317), *qCT6*.*6* (between RM3 and RM439), *qCT9*.*7* (between RM278 and RM160) and *qCT11*.*5* (between RM457 and RM21). Three of these large-effect CT QTL (*qCT4*.*6*, *qCT6*.*6* and *qCT11*.*5*) were also reported previously [4, 7, 15], while the others were new ones. In our phenotyping experiments [22], we found virtually all selected ILs showed significantly increased height, suggesting most, if not all, these CT QTLs might be involved in GA-related pathways.

## Discussion

In *Geng* breeding efforts for improved CT at the reproductive stage, donors for CT are almost exclusively *Geng* varieties that are known to be more tolerant to cold than *Xians*. However, we previously demonstrated that *Xian* varieties could be used as donors for improving CT in *Geng* varieties [22]. In fact, the *Xian* donors in three of the five populations used in this study were from Vietnam, India and south China, and are known to have poor CT. Interestingly, they all appeared to be good donors for CT as far as the number of selected CT progeny concerned. This was true for tolerances to other abiotic stresses such as drought, salinity and submergence in most BC breeding populations involving a diverse set of donors and recipients [19–21, 23]. We have proposed that future rice improvement requires successful integration of trait improvement and gene/QTL discovery [24, 31, 32], which can be achieved by examining how phenotypic selection was operating on the genetic variation of complex traits in the process of breeding. Thus, this study presents another case in our efforts for simultaneous improvement and genetic dissection of complex traits by marker-facilitated characterization of donor introgression in selected ILs. Moreover, when compared with the previous reports [3, 25, 29], the inclusion of random populations in this study provided additional evidence to justify the strategy of selective introgression. Several interesting findings regarding the real power of this strategy and the genetic basis of CT at the reproductive stage in rice worth more discussion.

The first one was the genomewide under-introgression of the donor genomes in both the random and selected ILs in the CY1 (*Geng*) genetic background as compared with the Mendelian expectation, which was in contrast to the general genomewide over-ingression in ILs of several *Xian* genetic backgrounds [2, 25]. This characteristic introgression patterns with the two subspecific genetic backgrounds of rice appeared to be consistent with the fact that *Geng* genomes have smaller pan genome size but larger core genome than *Xian* genomes [1], and with our observation that *Geng* genomes have much higher “genetic load” than the Xian genomes [33]. This also suggests that the overall level of donor introgression may also be characteristic of specific genetic backgrounds. Nevertheless, this genomewide under-introgression actually increased the power in detecting CT QTLs by selective introgression. Thus, the detection of 37 genomic regions harboring CT loci or an average nine FGUs (7 loci and 2 AGs) per population was highly efficient (Table 5, Fig 3) with the average 16.8 ILs per population. This was in contrast to the extremely low power in QTL detection using the random populations of larger size. Moreover, most identified CT loci were detected with P<0.0001 and 45% of the loci were detected in two or more populations, suggesting the rate of false positives, if any, was low in this study. This was not surprising because the 84 CT ILs were selected from the original 5 BC populations of 2,250 BC_2_F_2_ plants.

Secondly, we observed strong non-random associations between or among some of the detected CT loci, primarily detected as AGs each consisting of two or more unlinked but perfectly associated loci in the selected CT ILs from a single population. In these cases, the donor alleles at all loci of an AG were the target of the strong phenotypic selection for CT. The strongest one was branch C-1 that included 6 unlinked but highly associated loci of high introgression. In fact, 18 of the 22 CT ILs from population C (CY1/Fengaizhan) had the introgressed donor alleles at most C-1 loci. Theoretically, pronounced non-random associations between or among unlinked loci in selected progeny of a bi-parental cross suggest the presence of strong epistasis among genes acting in a same positively regulated pathway that leads to the target trait (CT) under strong directional selection [34]. If so, branch C-1 might represent an important pathway for CT in rice because ILs of this population showed the highest level of CT (Table 1). Our observation for the presence of several major branches in the CT genetic networks suggests the presence of several positively regulated pathways controlling CT in rice. Although it remains very challenging to determine what specific pathways were implicated by these putative QTL branches or associated QTL groups, transcriptome analyses using the CT ILs and its recipient under stress and non-stress conditions should provide valuable information in this respect [35–39].

Clearly, the putative genetic networks detected in this study should be verified. While it would be very difficult to clone and verify the molecular functions of the detected CT loci individually using the classical approaches of map-based cloning or of molecular biology, it is not difficult to confirm the putative CT genetic networks genetically. A straightforward strategy is to break, by recombination, each identified network or AG into individual loci and evaluate their effects individually in the progeny derived from the crosses between different ILs or between crosses between the ILs and CY1. Also, various omic tools and bioinformatic analyses can be very powerful to gain useful information for inferring the possible molecular mechanisms of the detected genetic networks by assessing the genomewide responses at different levels of carefully selected progeny from these crosses to cold stress [24, 34, 40]. Thus, the CT ILs developed in this study provide valuable materials for genetic and molecular dissection of complex genetic networks underlying complex traits such as CT in rice.

Our results have important implications for improving complex traits of *Geng* rice. The *Geng* rice gene pool is known to have very limited genetic diversity [1, 41]. Our result that all four *Xian* donors contributed CT enhancing alleles at quite different loci to the *Geng* recipient, CY1, suggested that there is a rich source of hidden genetic diversity in the *Xian* gene pool for improving CT of *Geng* rice. Thus, our strategy of using BC breeding, strong phenotypic selection plus genetic tracking and characterization using DNA markers provides a good solution for broadening the genetic diversity in the *Geng* gene pool. Thus, genetic complementarity provides an appropriate explanation for the hidden diversity and transgressive segregation of CT observed in this study and other complex traits in rice [19–21]. Finally, the CT FGUs and their relationships identified in the CT ILs provide useful genetic information for further improving rice CT and simultaneous verification of the identified CT genetic networks using pyramiding populations derived from crosses between ILs carrying independent CT FGUs from different donors.

## Conclusions

Total of 17 QTLs for CT were detected in 84 cold-tolerant ILs selected from five BC2 populations in CY1 genetic background using a consensus linkage map, three of which (*qCT3*.*12*, *qCT6*.*7* and *qCT9*.*6*) were validated in random BC populations. In addition, 46 functional genetic units (FGUs) including 37 single loci and 9 association groups for CT were detected by χ^2^ tests and multi-locus linkage disequilibrium analyses. The findings reported herein may be useful for knowledge-based rice improvement of CT. Future research will focus on validating the effects of these putative genetic networks detected in this study. Our results demonstrated that selective introgression is powerful in simultaneous improvement and genetic dissection of complex traits such as CT in rice.

## Supporting information

S1 Table QTLs for cold tolerance reported in previous studies. (DOCX)

S2 Table Summary statistics of ILs and recipient parent (Chaoyou1, CY1) for spikelet fertility (SF) selected under cold water stress. (DOCX)
