# Supplementary material for: QTL and QTL networks for cold tolerance at the reproductive stage detected using selective introgression in rice
Source: PLoS One. 2018 Sep 17;13(9):e0200846. doi: 10.1371/journal.pone.0200846 (PMC6141068; doi:10.1371/journal.pone.0200846)
Supplement: S1 Table — (DOCX) [file pone.0200846.s001.docx]

**Supplementary Table S1.** QTLs for cold tolerance reported in previous studies.

| Population | Traits | QTL | Chr | Marker | Reference |
| --- | --- | --- | --- | --- | --- |
| 2428/3037 | SF | *Ste1* | 1 | RZ154-R2635 | Liu et al. 1997 |
| 161 F_2_ | SF | *Ste3* | 1 | RZ566-RG236 |  |
|  | SF | *Ste2* | 12 | C362-C104 |  |
| Norin-PL8/Kiara397 | AL | *Qctb2* | 4 | RM317 | Saito et al. 2001 |
| 59 F_7_ |  | *Qctb1* | 4 | SCAB11-R740 |  |
| Koshihikari/Akihikari | SF | *qCT-1* | 1 | R1545 | Takeuchi et al. 2001 |
| 212 DHL | CL | *Qcl-1* | 1 | R1545 |  |
|  | SF | *QCT-7* | 7 | S778 |  |
|  | SF | *QCT-11* | 11 | C83 |  |
| Guichao/DXCWR | SF | *qSLT1-1* | 1 | RM81A | Liu et al. 2003 |
| 284 BC_4_F_2_ | SF | *qSLT2-1* | 6 | RM6 |  |
|  | SF | *qSLT8-2* | 8 | RM223 |  |
|  | SF | *qSLT9-1* | 9 | RM215 |  |
|  | SF | *qSLT11-2* | 11 | RM202 |  |
|  | SF | *qSLT11-1* | 11 | OSR1 |  |
| M-202/IR50 | SF | *qCTB2a* | 2 | RM324-RM301 | Andaya et al. 2003 |
| 191 RIL | SF | *Qctb3* | 3 | RM156-RM214 |  |
| Milyang/Hapcheonaengmi | CL | *cl1,clr1,pe1* | 1 | RM128-PBC121 |  |
| 80 RIL | CL | *clr9* | 9 | RM285-RM434 | Oh et al. 2004 |
|  | SF | *fer11* | 11 | RM3701-RM552 |  |
| Norin-PL8/Kiara397 49 NIL | AL | *Qctb1* | 4 | SCAB11-OSR15 | Saito et al. 2004 |
| KMXBG/Towada | SF | *QRCT7* | 7 | RM182-RM1789 | Dai et al. 2004 |
| 250 F_2_ | SF | *QRCT10* | 10 | G1010-RM239 |  |
| Hokkai-PL9/Hokkai287 288 BC_5_F_3_ | SF | *Qctb8* | 8 | RM5647-RM5434 | Kuroki et al. 2007 |
| KMXBG/Towada | SF | *QCTB-1-1* | 1 | RM1282-RM3148 | Xu et al. 2008 |
| BC_5_F_3_ | SF | *QCTB-4-1* | 4 | RM518-RM6770 |  |
|  | SF | *QCTB-4-2* | 4 | RM7200-RM821 |  |
|  | SF | *QCTB-5-1* | 5 | RM7452-RM7271 |  |
|  | SF | *QCTB-5-2* | 5 | RM19106-RM31 |  |
|  | SF | *QCTB-10-1* | 10 | RM3590-RM24918 |  |
|  | SF | *QCTB-10-2* | 10 | RM2125-RM2887 |  |
|  | SF | *QCTB-11-1* | 11 | RM1812-RM332 |  |
| Kirara397/Hatsushizuku | CT | *Qct1* | 1 | RM1003-RM3482 | Kuroki et al. 2009 |
| 114 RIL | CL | *Qcl2* | 2 | RM561-RM5427 |  |
|  | CL | *Qct10* | 10 | RM6691-RM333 |  |
| KMXBG/Towada | 10 traits | *Qctb1-1* | 1 | RM3148-RM6340 | Zeng et al. 2009 |
| 1525 NIL | 5 traits | *Qctb4-2,4-3,4-4* | 4 | RM5414-RM7200 |  |
|  | 10 traits | *Qctb4-1,4-5* | 4 | RM335-RM518 |  |
|  | 5 traits | *Qctb5-1* | 5 | RM7271-RM7452 |  |
| Norin-PL8/Kiara397 |  | *Ctb1* | 4 |  | Saito et al. 2010 |
| KMXBG/ZL1929-4 F_2_-F_3_ | SF | *Qctb7* | 7 | RM182-RM1132 | Zhou et al. 2010 |
| IR66160-121-4-4-2/Geumobyeo | SF | *QPSST-3* | 3 | RM569-RM231 | Suh et al. 2010 |
| 153 RIL | SF | *QPSST-7* | 7 | RM3767-RM1377 |  |
|  | SF | *QPSST-9* | 9 | RM24427-RM24545 |  |
| Reizip/Lijianggeihu BC_2_F_1_ | SF | *Qltspkst10.1* | 10 | S10010.9-S10014.4 | Ye et al. 2010 |
| Dasanbyeo/TR22183 | SF | *QTL2.1* | 2 | RM318-RM250 | Jiang et al. 2011 |
| 152 RIL | SF | *QTL8.1* | 8 | S08052-S08055 |  |
|  | SF | *QTL10.1* | 10 | S10001B-S10019 |  |
| J502/Hoshimar | SF |  | 3 | RM3180 | Mori et al. 2011 |
| 144 F_3_ | SF |  | 4 | RM5953 |  |
|  | SF |  | 11 | RM5824 |  |
| Lijiangxintuanheigu/Ukei 840 | SF | *Qltb3* | 3 | RM7000 | Shirasawa et al. 2012 |
| 192 F_2_ | SF | *Qltb3* | 3 | C11223-24.4 |  |
| Rice cultivars | SF |  | 1 | RM220 | Cui et al. 2013 |
| 327 | SF |  | 3 | RM571 |  |
|  | SF |  | 4 | RM336 |  |
|  | SF |  | 11 | RM21 |  |
| Suisei/Eikei88223 | SF | *qCTF7* | 7 | RM20923-RM5711 | Shinada et al. 2013 |
| 77 BIL | SF | *Qctf8* | 8 | RM22613-RM22755 |  |
|  | SF | *qCTF12* | 12 | RM5282-RM28661 |  |
| Rice cultivars | SF | *Qsfc1* | 1 | RM3475 | Suh et al. 2013 |
| 23 cultivars | SF | *Qsfc2* | 2 | RM5764 |  |
|  | SF | *Qsfc7* | 7 | RM481 |  |
|  | SF | *Qsfg8* | 8 | RM256 |  |
|  | SF | *Qsfg8* | 8 | RM447 |  |
|  | SF | *Qsfg9* | 9 | RM1026 |  |
|  | SF | *Qsfg10* | 10 | RM590 |  |
|  | SF | *Qsfg12* | 12 | RM512 |  |
| NJ/DX 151 BC_2_F_1_ | PSR | *qRC10-2* | 10 | RM25570-RM304 | Xiao et al. 2014 |
| HHZ-ILs 497 BC_1_F_5_ |  | *qCT-3-2* | 3 | - | Zhu et al. 2015 |
| 174 Chinese rice accessions | SF | *qLTSSR1-2* | 1 | RM1095 | Pan et al. 2015 |
|  |  | *qLTSSR1-3* | 1 | TC56 |  |
|  |  | *qLTSSR1-1* | 1 | RM23 |  |
|  |  | *qCTSSR1-2* | 1 | RM1151 |  |
|  |  | *qCTSSR1-1* | 1 | RM5496 |  |
|  |  | *qLTSSR2-1* | 2 | RM1255 |  |
|  |  | *qCTSSR2-1* | 2 | RM3220 |  |
|  |  | *qLTSSR3-1* | 3 | RM3126 |  |
|  |  | *qCTSSR3-1* | 3 | RM143 |  |
|  |  | *qLTSSR4-2* | 4 | RM1112 |  |
|  |  | *qLTSSR4-1* | 4 | RM3217 |  |
|  |  | *qCTSSR4-1* | 4 | RM1153 |  |
|  |  | *qLTSSR5-1* | 5 | RM2494 |  |
|  |  | *qLTSSR6-1* | 6 | RM111 |  |
|  |  | *qCTSSR6-2* | 6 | RM190 |  |
|  |  | *qCTSSR6-3* | 6 | TC148 |  |
|  |  | *qCTSSR6-1* | 6 | RM253 |  |
|  |  | *qLTSSR8-2* | 8 | RM223 |  |
|  |  | *qLTSSR8-1* | 8 | RM126 |  |
|  |  | *qCTSSR8-1* | 8 | RM126 |  |
|  |  | *qCTSSR9-1* | 9 | RM242 |  |
|  |  | *qLTSSR10-1* | 10 | RM7361 |  |
|  |  | *qCTSSR11-1* | 11 | RM6544 |  |
|  |  | *qCTSSR12-1* | 12 | RM1227 |  |
| Kuchum/Hitomebore BC_6_F_4_ | SF | *qCT-4* | 4 | RM5687-2–S38P21-1 | Endo et al. 2016 |
| Chiyohonami/BI6-12 F_2_ | SF |  | 1 | RM8068–RM8146 |  |
|  | SF |  | 4 | RM4835–S72D21-1 |  |
| 400 *O. sativa* accessions | SF | *qFERCT2* | 2 | - | Shakiba et al. 2017 |
|  |  | *qFERCT6-1* | 6 | - |  |
|  |  | *qFERCT6-2* | 6 | - |  |
|  |  | *qFERCT6-3* | 6 | - |  |
|  |  | *qFERCT6-4* | 6 | - |  |
|  |  | *qFERCT7* | 7 | - |  |
|  |  | *qFERCT12* | 12 | - |  |
| KMXBG/Towada BC_6_F_2_ | SF | *qCTB4-1* | 4 | RM16349-SSR29 | Zhang et al. 2017 |
| NIL1913/Towada 3102 F_2_ | SF |  |  |  |  |
| NIL1913/Towada 179 F_2_ | SF |  |  |  |  |
| KMXBG/Towada BC_7_F_5_ | SF | *qCTB10 - 2* | 10 | RM25121-MM0568 | Li et al. 2018 |
