# Supplementary material for: QTL and QTL networks for cold tolerance at the reproductive stage detected using selective introgression in rice
Source: PLoS One. 2018 Sep 17;13(9):e0200846. doi: 10.1371/journal.pone.0200846 (PMC6141068; doi:10.1371/journal.pone.0200846)
Supplement: S2 Table — (DOCX) [file pone.0200846.s002.docx]

**Supplementary Table S2.** Summary statistics of ILs and recipient parent (Chaoyou1, CY1) for spikelet fertility (SF) selected under cold water stress.

| Donor parent | Cross  code | N1 ^1^ | SF in 2008 | | |  | SF in 2009 | | |
| --- | --- | --- | --- | --- | --- | --- | --- | --- | --- |
|  |  |  | N2 ^2^ | Mean (%) | Range (%) |  | N3 ^3^ | Mean (%) | Range (%) |
| X22 | A | 450 | 28 | 65.6 | 50.7-87.3 |  | 19 | 57.7 | 40.8-79.4 |
| Yuanjing7 | B | 450 | 41 | 71.1 | 50.0-90.1 |  | 25 | 71.7 | 52.6-84.2 |
| Fengaizhan | C | 450 | 44 | 74.1 | 52.2-98.5 |  | 43 | 72.1 | 53.2-86.1 |
| Chhomrong | D | 450 | 24 | 75.6 | 51.4-87.8 |  | 20 | 68.5 | 45.6-83.4 |
| Doddi | E | 450 | 25 | 71 | 50.2-90.0 |  | 25 | 65.4 | 48.7-80.6 |
| Chaoyou1 | CY1 |  |  | 24.8 | 19.0-30.0 |  |  | 35.1 | 33.7-36.5 |

^1^ N1 is the original size of the BC_2_F_4_ population used for screening CT at the reproductive stage.

^2^ N2 is the number of selected plants with SF>50% from each population in 2008.

^3^ N3 is the number of selected ILs with SF>40% from each population in 2009.
